# Clinical application of fetal Nuchal Translucency combined with noninvasive prenatal testing in screening chromosome abnormalities

**DOI:** 10.1371/journal.pone.0344739

**Published:** 2026-03-16

**Authors:** Guangzhen Fu, Shuang Hu

**Affiliations:** 1 Department of Clinical Laboratory, The First Affiliated Hospital of Zhengzhou University, Key Clinical Laboratory of Henan Province, Zhengzhou, China; 2 Genetic and prenatal diagnosis center, The First Affiliated Hospital of Zhengzhou University, Zhengzhou, China; Universita degli Studi di Roma Tor Vergata, ITALY

## Abstract

**Objective:**

To explore the clinical application of fetal nuchal translucency (NT) combined with noninvasive prenatal testing (NIPT) in screening for fetal chromosomal diseases.

**Methods:**

A retrospective analysis was performed on NIPT results, prenatal diagnoses, and pregnancy outcomes of early pregnant women with enlarged NT at our center from 01/07/2018 to 01/01/2021. To ensure data integrity and minimize extraction bias, phased data access was adopted: the first batch of data was extracted from 01/07/2019 (start of Phase 1) to 20/07/2019 (end of Phase 1), covering participants examined from 01/07/2018 to 30/06/2019; the second batch was extracted from 01/02/2020 (start of Phase 2) to 20/02/2020 (end of Phase 2), including participants examined from 01/07/2019 to 01/01/2020. Chi-square tests were used to compare the incidence of fetal chromosomal abnormalities between the enlarged NT and normal NT groups, as well as among the subgroups of enlarged NT.

**Results:**

Out of 48,130 women screened by NIPT, 774 had enlarged NT (≥2.5 mm); among these, 85 were classified as high-risk, and 72 underwent invasive diagnosis, resulting in 64 positive cases (including 42 cases of trisomy 21, among others). The thickening of NT was correlated with a higher detection rate of abnormalities, which was statistically significant. Among the 689 cases of enlarged NT with low-risk NIPT results, 20 were lost to follow-up, 5 terminated their pregnancies, while the others had healthy live births.

**Conclusion:**

Early pregnancy NT combined with NIPT demonstrates a high predictive value for fetal chromosomal abnormalities and holds significant importance for clinical pregnancy guidance.

## Introduction

Fetal chromosomal abnormalities significantly contribute to developmental disorders and miscarriages. Common conditions include trisomy 21 (Down syndrome), trisomy 18 (Edwards syndrome), and trisomy 13 (Patau syndrome) [1]. These diseases seriously affect patients’ growth and development and impose significant psychological and financial burdens on their families [2]. Chromosomal abnormalities have a high incidence in China as well as in the world, and effective treatments are currently lacking. Therefore, it is particularly important to identify fetuses with chromosomal abnormalities early through prenatal screening and diagnosis, thereby reducing birth defects and improving the health quality of the population.

In recent years, the application of fetal nuchal translucency (NT) measurement and non-invasive prenatal testing (NIPT) in the screening of fetal chromosomal diseases has attracted extensive attention [3]. As an ultrasonic soft marker, the size of NT is usually correlated with congenital structural abnormalities of the fetus and also has diagnostic value for fetal chromosomal abnormalities. However, relying solely on NT to determine the presence or absence of fetal chromosomal abnormalities is insufficient in evidence, and other prenatal testing methods are required [4]. Non-invasive prenatal testing is a method that uses cell-free fetal DNA in maternal plasma to detect common fetal chromosomal aneuploidies. It has been widely used worldwide due to its advantages such as safety, non-invasiveness, high accuracy, and large throughput [5]. NIPT is a highly efficient screening method for 21/18/13-trisomy, and has been adopted as a first-line or contingency screening protocol globally since 2012 [6]. Therefore, the combined application of NT and NIPT may further improve the screening efficiency.

In summary, the combined detection of fetal nuchal translucency (NT) and non-invasive prenatal testing (NIPT) provides new ideas and methods for the screening of fetal chromosomal diseases, and can offer reliable basis for early intervention and management. In-depth research on these two methods can provide more effective screening strategies for clinical practice, improve fetal health outcomes, and reduce family burdens. This study aims to explore the clinical application value of combined NT and NIPT detection in the screening of fetal chromosomal diseases.

## Materials and methods

### Clinical data

A total of 48,130 pregnant women who underwent non-invasive prenatal testing (NIPT) at the Genetic Center of our hospital from 01/07/2018 to 01/01/2021 were selected as the research subjects. A retrospective analysis was performed on the screening results, prenatal diagnosis results, and pregnancy outcomes of pregnant women who underwent NIPT due to increased nuchal translucency (NT). To ensure data integrity and minimize extraction bias, phased data access was adopted: the first batch of data was extracted from 01/07/2019 (start of Phase 1) to 20/07/2019 (end of Phase 1), covering participants examined from 01/07/2018 to 30/06/2019; the second batch was extracted from 01/02/2020 (start of Phase 2) to 20/02/2020 (end of Phase 2), including participants examined from 01/07/2019 to 01/01/2020. Data quality checks were performed after each extraction to verify completeness, consistency, and accuracy of key variables. The age of the pregnant women ranged from 19 to 45 years, with an average age of 29.3 years. The gestational age was 12–22 + 6 weeks, with an average of (18.5 ± 1.3) weeks. Inclusion criteria: NT ≥ 2.5 mm as the cut-off value, without other ultrasonic abnormalities. Among 72 pregnant women with high-risk NIPT results, 25 underwent chorionic villus sampling, and 47 underwent amniocentesis for confirmation. Among those with increased NT but low-risk NIPT results, 5 cases chose induced abortion due to severe structural malformations during pregnancy. All pregnant women were informed of the details and signed the informed consent form. This study was approved by the Medical Ethics Committee of the hospital (2018-YB-08).

## Methods

### NT measurement

The GE Voluson E8 color Doppler ultrasound diagnostic instrument with a convex probe (frequency: 3.0–5.0 MHz) was used. The thickness of fetal nuchal translucency (NT) in the first trimester was measured at 11–13 + 6 weeks of gestation. NT ≥ 2.5 mm was considered suspiciously increased, and NT ≥ 3 mm was defined as increased.

NIPT detection
  10 ml of peripheral venous blood was collected from pregnant women into non-invasive special blood collection tubes. Cell-free DNA was extracted from maternal blood to prepare sequencing libraries. After library quantification, high-throughput sequencing was performed using a gene sequencer (Nextseq CN500, USA). The obtained gene sequences were compared with the human reference genome, and finally the risk of fetal chromosomal aneuploidy and copy number variations (CNVs) was determined. The risk value was evaluated by Z-score.

### Invasive prenatal diagnosis

When NIPT results indicated high risk, pregnant women signed the informed consent form and underwent chorionic villus sampling (at 11–13 + 6 weeks of gestation) to obtain approximately 10 mg of chorionic villus tissue or amniocentesis (at 17–24 weeks of gestation) to obtain about 13 ml of amniotic fluid.

### CNV-seq analysis

Genomic DNA was extracted from 25 cases of chorionic villus tissue, 7 cases of amniotic fluid cells, and 5 cases of abortion specimens. Restriction endonucleases were used to hydrolyze DNA into small fragments (approximately 200 bp in size) for library preparation. After library quantification, high-throughput sequencing was performed using a high-throughput sequencer (Nextseq CN500, USA). After sequencing, the results were compared and analyzed with the human genome. A dedicated algorithm was used to obtain CNVs for each sample. The clinical significance of each CNV was interpreted with reference to the latest ACMG guidelines.

### Chromosomal karyotype analysis

Approximately 13 ml of each amniotic fluid sample (40 cases in total) was centrifuged at 1500 r/min for 8 minutes. The supernatant was discarded, and the precipitate was used for amniotic fluid cell culture. After cell harvesting, G-banding was performed, and karyotype analysis was conducted according to the ISCN (2020) standards.

### Follow-up

The pregnancy outcomes of all pregnant women who underwent invasive prenatal diagnosis were followed up by telephone.

### Statistical analysis

Graphpad Prism software was used for statistical analysis. Count data were expressed as percentages, and the chi-square test was used for comparison between groups. A P value < 0.05 was considered statistically significant.

## Results

### Positive Screening Rate of Fetal Chromosomal Abnormalities

Among 48,130 pregnant women who underwent NIPT screening, 774 cases had NT thickening. Among them, 85 cases had positive screening results, with a positive detection rate of 10.98% (85/774). Compared with the positive detection rate of 3.26% (1542/47356) in the normal NT group, the difference was statistically significant (χ² = 139.2, P < 0.0001). Compared with the positive detection rate of 3.38% (1627/48130) in the simple NIPT group, the difference was also statistically significant (χ² = 130.3, P < 0.0001). Among the 85 positive cases, 64 cases were confirmed as true positives by amniotic fluid karyotype analysis or CNV-seq analysis, and 8 cases were false positives. The detection rate of chromosomal abnormalities was 8.26% (64/774). The positive predictive value was 88.88% (64/72). There were 689 cases with NT thickening but negative NIPT screening results, among which 5 cases had miscarriages, and the results of CNV-seq analysis of the abortuses were all normal. Details are shown in Table 1.

**Table 1:** The screening results for fetal chromosomal abnormalities.

Screening results	Total samples	Screened positive	Positive detection rate	True positive	PPV%	False positive	Lost to follow-up
NT thickening	774	85	10.98%	64	75.29	8	13
Normal NT	47356	1542	3.26%	910	59.01	452	176
Total NIPT	48130	1627	3.38%	974	59.86	464	189

### Fetal Karyotype/CNV-seq results of invasive prenatal diagnosis

Among the 85 cases with NT thickening and positive NIPT screening results, 64 cases were true positives, including 42 cases of trisomy 21 (accounting for 65.6%), 6 cases of trisomy 18, 3 cases of trisomy 13, 9 cases of sex chromosome abnormalities, and 4 cases of microdeletion/microduplication. Details are shown in Table 2.

**Table 2:** The karyotype/CNV-seq results of the fetus undergoing invasive prenatal diagnosis.

Chromosome abnormalities	Number of cases
47,XN, + 21	42
47,XN, + 13	3
47,XN, + 18	6
45,X	5
47,XXX	3
48,XXYY	1
Microdeletion/microduplication	4
False positive	8
Lost to follow-up	13
Total number	85

### Comparison of NT Thickness and occurrence of chromosomal abnormalities

The 774 fetuses with NT thickening were divided into 5 groups according to different NT values. It was found that with the increase of NT thickness, the positive detection rate of NIPT screening and the detection rate of fetal chromosomal abnormalities gradually increased. The difference in the detection rates of fetal chromosomal abnormalities among groups with NT thickening was statistically significant (χ² = 31.87, P < 0.0001). The first group, with NT thickening but unknown specific values, was listed separately, with a positive detection rate of 9.57%, close to the overall positive detection rate of 10.98%. The positive predictive value also showed an upward trend with the increase of NT value. Details are shown in Table 3.

**Table 3:** Comparison of the incidence of fetal chromosomal abnormalities in each group with NT thickening.

Group of NT test result (mm)	Number of samples	Screened positive samples	Positive detection rate	True positive	Abnormality rate	False positive	PPV%
NT thickening with unknown value	94	9	9.57%	8	8.50%	1	88.8
2.5 ≤ NT ＜ 3	365	26	7.12%	18	4.90%	4	81.8
3 ＜ NT ≤ 4	259	34	13.12%	24	9.26%	2	92.3
4 ＜ NT ≤ 5	44	10	22.72%	8	18.18%	1	88.8
5 ≤ NT	12	6	50%	6	50%	0	100.0
Total	774	85	10.98%	64	8.26%	8	88.8

### Pregnancy outcomes

Among the 85 pregnant women with positive screening results, 64 cases were confirmed as true positives by invasive prenatal diagnosis and all chose to terminate the pregnancy; 8 cases were false positives, all of whom had normal pregnancies until delivery, and no abnormalities were found after birth; 13 cases were lost to follow-up. Among the 689 cases with NT thickening but low-risk NIPT results, 5 cases had miscarriages, 20 cases were lost to follow-up, and the remaining fetuses were born healthy (664/689).

## Discussion

Fetal nuchal translucency (NT) is the accumulation of fluid under the skin at the back of the fetal neck during 11–14 weeks of pregnancy, and its thickness can be measured by ultrasound. Changes in NT thickness are closely related to the fetal physiological and developmental status. Normally, NT thickness is measured between 11 and 14 weeks of gestation and increases with gestational age. It is generally believed that the normal range of NT is less than 3.5 mm, while a thickness of ≥3.0 mm indicates a potential risk of fetal abnormalities, such as chromosomal abnormalities and structural malformations [7]. Studies have shown that NT thickness is significantly correlated with the incidence of fetal dysplasia, congenital heart defects and other diseases, making NT one of the important indicators for early screening [8].

Compared with serological Down syndrome screening, the emergence of NIPT has greatly improved the accuracy of screening. Conventional NIPT mainly screens for aneuploidies of 21/13/18 trisomies and sex chromosomes. Expanded NIPT can screen for common fetal chromosomal aneuploidies, large chromosomal deletions/duplications greater than 10Mb, and relatively high-incidence microdeletion diseases greater than 3Mb and located in specific syndrome-related chromosomal fragment positions.

This study found that 1.6% (774/48130) of fetuses in the pregnancy population had NT thickening (NT ≥ 2.5 mm), which was higher than the 0.96% in early foreign studies [9]. In 2013, domestic scholars collected data of 14881 pregnant women and found that 118 cases had fetal NT thickening, with an incidence rate of 0.79% [10]. This discrepancy in findings may result from differences in the definitions and parameters used to define NT thickening. Therefore, accurately measuring NT thickness is crucial due to its strong correlation with fetal anomalies and chromosomal defects, establishing it as a vital marker in prenatal screening [11,12].

This study found that the combined positive detection rate of the two was 10.98%, which was significantly higher than the positive detection rate of those with normal NT and the positive detection rate of NIPT alone. Therefore, the combination of the two can significantly improve the detection of fetuses with chromosomal abnormalities. After confirmation by invasive prenatal diagnosis, the confirmed rate of chromosomal abnormalities in the combined positive cases was 8.26% (64/774). As early as 1992, British scholars first proposed that increased fetal NT value is closely related to chromosomal abnormalities in their study of 827 cases [13]. Subsequent scholars have consistently elucidated the intricate relationship between nuchal translucency (NT) thickening and chromosomal abnormalities, highlighting the significance of this marker in prenatal diagnostics. Studies have demonstrated that chromosomal microarray analysis (CMA) serves as a critical tool for identifying chromosomal abnormalities that may not be detectable through conventional karyotyping, thereby enhancing the effectiveness of prenatal screenings for fetuses exhibiting increased NT measurements [14,15]. Furthermore, the detection rates of chromosomal abnormalities have been shown to escalate with the degree of NT thickening, underscoring the necessity of integrating advanced genetic testing methods in cases of elevated NT [16,17]. As research continues to evolve, the implications of elevated NT measurements in conjunction with chromosomal analysis are becoming increasingly pivotal in guiding clinical decision-making and improving prenatal care outcomes [18,19].

Our results are significantly different from the abnormal confirmation rates of other institutions and only similar to the results of some studies. The possible reasons are as follows: First, different studies have different definitions of NT thickening; second, the pregnant populations counted by different research centers are different; third, we did not perform invasive prenatal diagnosis on all cases with NT thickening, but only performed puncture for confirmation on pregnant women with positive NIPT screening results. The karyotype or CNV-seq results of fetuses with NT thickening but negative NIPT screening are unknown. Only 5 cases of aborted fetuses that had undergone induction of labor were subjected to genetic analysis. For the remaining cases, we only knew through follow-up that the fetuses were born healthy and no abnormalities have been found so far.

In this study, among the 64 fetuses with NT thickening and chromosomal abnormalities, there were 42 cases of trisomy 21 (65.6%) and 51 cases of trisomy syndrome (79.68%). In various studies, the frequency and types of chromosomal abnormalities associated with fetuses exhibiting increased nuchal translucency (NT) have shown significant variability, indicating the complexity and heterogeneity of this condition across different populations and methodologies employed in research. Notably, the American College of Obstetricians and Gynecologists and the Society for Maternal-Fetal Medicine have recommended genetic counseling and diagnostic testing for nuchal translucency measurements at or above 3.0 mm, regardless of previous negative screening results, emphasizing the clinical importance of accurately assessing NT values [8]. Furthermore, the presence of increased NT has been linked to a higher risk of atypical chromosomal abnormalities, with studies indicating that the likelihood of detecting significant chromosomal anomalies escalates in conjunction with elevated NT levels [12,20]. Overall, the diverse findings across different studies underscore the necessity for continued investigation into the implications of increased nuchal translucency and its association with chromosomal abnormalities in the context of prenatal screening and diagnosis. The proportion of trisomies in this study was significantly higher than that in other institutions. The reason may be that the pregnant women who underwent fetal chromosomal karyotyping in this study were all cases with NT thickening and positive NIPT screening, while other studies were cases with simple NT thickening. For those with NT thickening but negative NIPT screening, only 5 cases underwent induction of labor, and the CNV-seq analysis of their abortuses showed no abnormalities. This indicates that NT detection combined with NIPT screening can significantly improve the detection rate of trisomy syndrome. Moreover, fetal NT thickening is an important indicator for the occurrence of trisomy 21 syndrome.

NT thickening is not only related to chromosomal aneuploidy but also to CNVs. Many scholars have pointed out that CNVs analysis of fetuses with NT thickening and normal chromosomes found that the detection rate of clinically significant microdeletions and microduplications is 7.7%−8.3% [21,22]. In this study, the confirmed rate of CNV was 6.25% (4/64), indicating that NT thickening is indeed related to CNVs. Therefore, the combined application of the two is also beneficial to the detection of CNVs. However, not all cases with NT thickening in this paper underwent CNV-seq analysis, so there is a possibility of missed detection, which is a major limitation of this paper. At the same time, the overall sample size of chromosomal karyotype or CNV-seq analysis in this study is small, and there are some flaws in the collection of sample data, which may inevitably lead to bias in the results.

In this study, according to different NT values, the cases were divided into several groups. By comparing the differences in positive detection rates and the incidence of chromosomal abnormalities between groups, we concluded that with the increase of fetal NT value, the positive detection rate of NIPT gradually increases, and the incidence of fetal chromosomal abnormalities also shows an increasing trend. The positive predictive values are all higher than 80%, with an overall positive predictive value of 88.8%. Especially when the NT value is ≥ 5 mm, 50% (6/12) of the samples are screened positive, and the positive predictive value reaches 100. When the NT value is greater than 3 mm, the positive predictive value is close to 90, but there are still false positives. We found that the positive predictive value of combined screening (88.88%, 64/72) is much higher than that of NT screening alone (8.26%, 64/774). When NT is thickened but NIPT results are negative, most fetuses are born healthy (664/689, 96.37%), indicating that the combination of the two has a strong ability to rule out negative cases. In clinical genetic counseling, for pregnant women with increased NT but negative results from NIPT, it is recommended to continue the pregnancy and undergo regular prenatal check-ups. This recommendation is based on a comprehensive assessment of the health of the mother and fetus, aimed at ensuring timely monitoring of potential risks throughout the pregnancy and providing necessary support and interventions [23]. Regular prenatal check-ups can not only help doctors identify and address potential issues promptly but also enhance the mother’s confidence in her and her fetus’s health, promoting her psychological and emotional well-being [24]. In this way, pregnant women can receive more personalized and humane medical services, thereby improving the overall pregnancy experience and outcomes [25]. Although NT detection is an important indicator for fetal chromosomal abnormalities, its positive detection rate is low. NIPT has the advantage of high accuracy in chromosomal screening and can avoid the risk of miscarriage caused by invasive prenatal diagnosis, but if its indications and contraindications are not paid attention to, the results are likely to be inaccurate. In this study, we used the combination of NT and NIPT and found that their combination has high predictive value for fetal chromosomal abnormalities in early pregnancy, which is of great significance for clinical pregnancy guidance.

In conclusion, NT combined with NIPT has high clinical value in fetal chromosomal abnormalities, which can reduce the number of invasive prenatal diagnoses in the short term and is worthy of promotion in prenatal diagnosis. For those with NT thickening but negative NIPT, the pregnancy outcome is good. It is necessary to pay attention to regular prenatal check-ups to minimize the birth of congenitally abnormal children. We look forward to large-sample studies from various research centers to confirm our research results.

## Supporting information

S1 Fig A flow diagram detailing the selection and outcomes of the cohort. (PNG)

S1 Table The anonymized dataset necessary to replicate the study findings. (XLSX)

## References

[1] Akalin H, Sahin IO, Paskal SA, Tan B, Yalcinkaya E, Demir M, et al. Evaluation of chromosomal abnormalities in the postnatal cohort: A single-center study on 14,242 patients. J Clin Lab Anal. 2024;38(1–2):e24997. doi: 10.1002/jcla.24997 38115218 PMC10829689

[2] Stadler JA 3rd. Neurosurgical Evaluation and Management of Patients with Chromosomal Abnormalities. Neurosurg Clin N Am. 2022;33(1):61–5. doi: 10.1016/j.nec.2021.09.012 34801142

[3] Petersen OB, Smith E, Van Opstal D, Polak M, Knapen M, Diderich KEM. Nuchal translucency of 3.0-3.4 mm an indication for NIPT or microarray? Cohort analysis and literature review. Acta Obstet Gynecol Scand. 2020;99(6):765–74. 32306377 10.1111/aogs.13877 PMC7318216

[4] Bakker M, Pajkrt E, Bilardo CM. Increased nuchal translucency with normal karyotype and anomaly scan: what next?. Best Pract Res Clin Obstet Gynaecol. 2014;28(3):355–66. doi: 10.1016/j.bpobgyn.2013.10.004 24332983

[5] Lee DE, Kim H, Park J, Yun T, Park DY, Kim M, et al. Clinical Validation of Non-Invasive Prenatal Testing for Fetal Common Aneuploidies in 1,055 Korean Pregnant Women: a Single Center Experience. J Korean Med Sci. 2019;34(24):e172. doi: 10.3346/jkms.2019.34.e172 31222985 PMC6589404

[6] Riggs ER, Andersen EF, Cherry AM, Kantarci S, Kearney H, Patel A, et al. Technical standards for the interpretation and reporting of constitutional copy-number variants: a joint consensus recommendation of the American College of Medical Genetics and Genomics (ACMG) and the Clinical Genome Resource (ClinGen). Genet Med. 2020;22(2):245–57. doi: 10.1038/s41436-019-0686-8 31690835 PMC7313390

[7] Souka AP, Von Kaisenberg CS, Hyett JA, Sonek JD, Nicolaides KH. Increased nuchal translucency with normal karyotype. Am J Obstet Gynecol. 2005;192(4):1005–21. doi: 10.1016/j.ajog.2004.12.093 15846173

[8] Hui L, Pynaker C, Bonacquisto L, Lindquist A, Poulton A, Kluckow E, et al. Reexamining the optimal nuchal translucency cutoff for diagnostic testing in the cell-free DNA and microarray era: results from the Victorian Perinatal Record Linkage study. Am J Obstet Gynecol. 2021;225(5):527.e1-527.e12. doi: 10.1016/j.ajog.2021.03.050 33957116

[9] Maya I, Yacobson S, Kahana S, Yeshaya J, Tenne T, Agmon-Fishman I, et al. Cut-off value of nuchal translucency as indication for chromosomal microarray analysis. Ultrasound Obstet Gynecol. 2017;50(3):332–5. doi: 10.1002/uog.17421 28133835

[10] Sun L, Wang X, Wu Q, Ruan Y, Yao L. Value of nuchal translucency thickening in the fetal chromosome abnormality screening. Zhonghua Fu Chan Ke Za Zhi. 2013;48(11):819–23. 24444557

[11] Montaguti E, Rizzo R, Diglio J, Di Donna G, Brunelli E, Cofano M, et al. Increased nuchal translucency can be ascertained using transverse planes. Am J Obstet Gynecol. 2022;227(5):750.e1-750.e6. doi: 10.1016/j.ajog.2022.05.057 35662633

[12] Bagde ND, Bagde M, Lone Z, Agrawal S, Nayak P, Pati SK. Effect of Exogenous Progesterone on Fetal Nuchal Translucency: An Observational Study. Cureus. 2022;14(12):e33023. 10.7759/cureus.33023 PMC9883054 36721559

[13] Nicolaides KH, Azar G, Byrne D, Mansur C, Marks K. Fetal nuchal translucency: ultrasound screening for chromosomal defects in first trimester of pregnancy. BMJ. 1992;304(6831):867–9. 1392745 10.1136/bmj.304.6831.867 PMC1882788

[14] Shi Y, Li X, Ju D, Li Y, Zhang X, Zhang Y. Abnormal chromosomes identification using chromosomal microarray. J Obstet Gynaecol. 2022;42(6):2025–32. doi: 10.1080/01443615.2022.2074786 35659171

[15] Lan L, Luo D, Lian J, She L, Zhang B, Zhong H, et al. Chromosomal Abnormalities Detected by Chromosomal Microarray Analysis and Karyotype in Fetuses with Ultrasound Abnormalities. Int J Gen Med. 2024;17:4645–58. doi: 10.2147/IJGM.S483290 39429961 PMC11488349

[16] Chen M-J, Lü A F-T, Huang P, Zhao Q. Chromosomal structural abnormalities in men with semen abnormality and analysis of 19 cases of first-reported abnormal karyotype. Zhonghua Nan Ke Xue. 2022;28(5):402–7. 37477478

[17] Zheng J, Wang T, Sun H, Guan Y, Yang F, Wu J, et al. Genetic correlation between fetal nuchal translucency thickening and cystic hygroma and exploration of pregnancy outcome. Sci Rep. 2024;14(1):27191. doi: 10.1038/s41598-024-76628-y 39516223 PMC11549315

[18] Samura O, Nakaoka Y, Miharu N. Sperm and oocyte chromosomal abnormalities. Biomolecules. 2023;13(6). 10.3390/biom13061010 PMC10296299 37371589

[19] Kagan KO, Sonek J, Kozlowski P. Antenatal screening for chromosomal abnormalities. Arch Gynecol Obstet. 2022;305(4):825–35. doi: 10.1007/s00404-022-06477-5 35279726 PMC8967741

[20] Zhou F, Yang M, Zhang Z, Zhu H, Wang J, Li L, et al. Evaluating the clinical utility and strategy of whole-exome sequencing testing for fetuses with increased nuchal translucency. Am J Obstet Gynecol. 2025;233(5):483.e1-483.e10. doi: 10.1016/j.ajog.2025.05.003 40381797

[21] Lund ICB, Christensen R, Petersen OB, Vogel I, Vestergaard EM. Chromosomal microarray in fetuses with increased nuchal translucency. Ultrasound Obstet Gynecol. 2015;45(1):95–100. doi: 10.1002/uog.14726 25393210

[22] Konialis C, Pangalos C. Dilemmas in prenatal chromosomal diagnosis revealed through a single center’s 30 years’ experience and 90,000 cases. Fetal Diagn Ther. 2015;38(3):218–32. 25659342 10.1159/000368604

[23] Schaa KL, Biesecker BB. Where is the “counseling” in prenatal genetic counseling?. Patient Educ Couns. 2024;124:108278. 38593481 10.1016/j.pec.2024.108278

[24] Lahiri S, Mersch J, Zimmerman J, Mauer Hall C, Moriarty K, Gemmell A, et al. Randomized control trial comparing genetic counseling service delivery models in an underserved population. J Genet Couns. 2025;34(2):e1975. doi: 10.1002/jgc4.1975 39370944 PMC11866741

[25] McGraw E, Rispoli J, Horner MB, Heiman GA. Genetic counseling certificate program: A program evaluation of undergraduate exposure to genetic counseling. J Genet Couns. 2022;31(4):1003–7. doi: 10.1002/jgc4.1564 35194893 PMC9541149

