# Supplementary figures and images for: Clinical application of fetal Nuchal Translucency combined with noninvasive prenatal testing in screening chromosome abnormalities
Source: PLoS One. 2026 Mar 16;21(3):e0344739. doi: 10.1371/journal.pone.0344739 (PMC12991258; doi:10.1371/journal.pone.0344739)

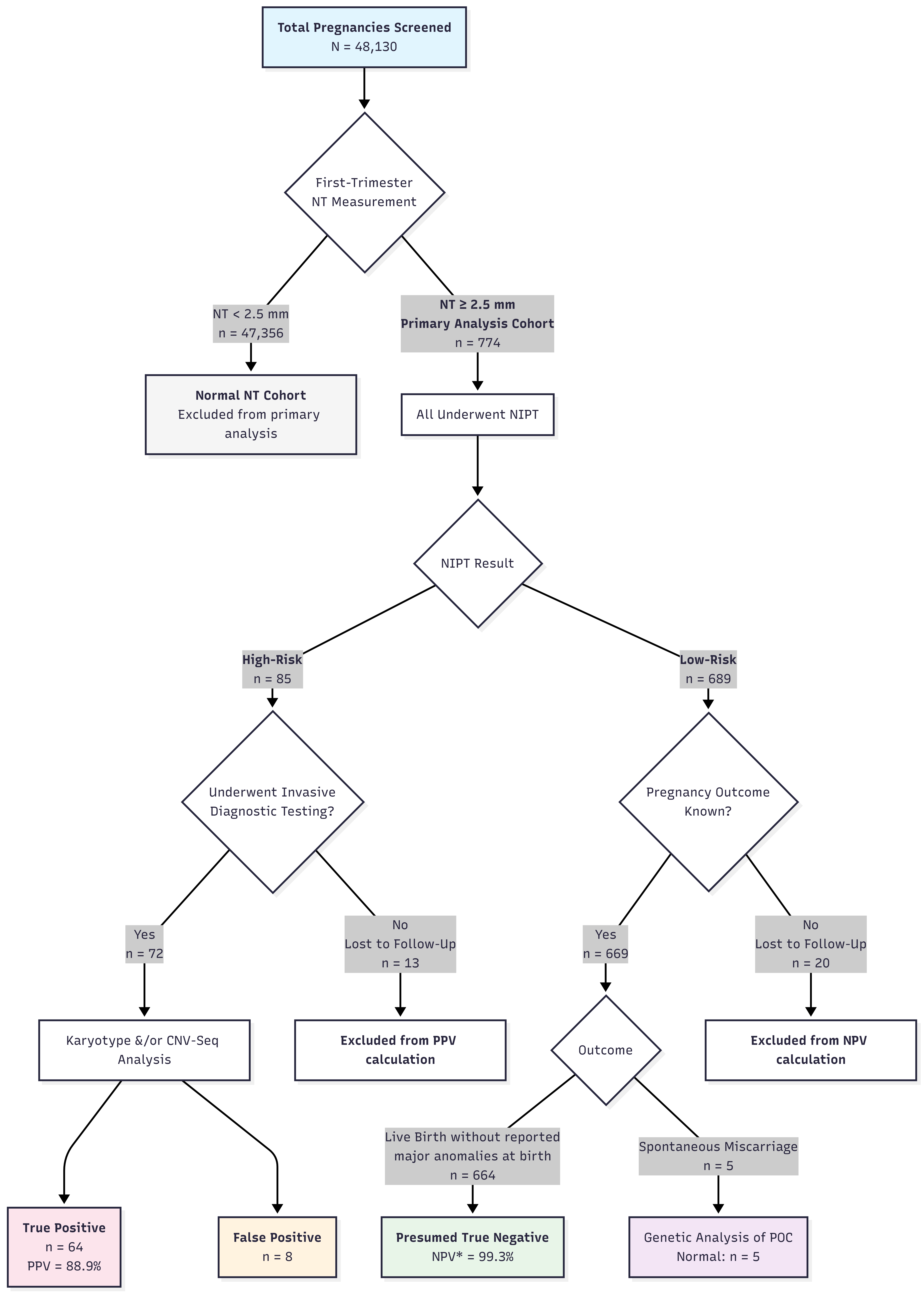

Supplement: S1 Fig — (PNG) [file pone.0344739.s001.png]
